# The use of three-dimensional endoscope in transnasal skull base surgery: A single-center experience from China

**DOI:** 10.3389/fsurg.2022.996290

**Published:** 2022-09-23

**Authors:** Guo Xin, Yajing Liu, Yicheng Xiong, Shenhao Xie, Hai Luo, Liming Xiao, Xiao Wu, Tao Hong, Bin Tang

**Affiliations:** ^1^ Department of Neurosurgery, The First Affiliated Hospital of Nanchang University, Nanchang, China; ^2^ Operating Theater, The First Affiliated Hospital of Nanchang University, Nanchang, China

**Keywords:** three-dimensional endoscope, endoscopic endonasal surgery, skull base surgery, depth perception, stereo vision

## Abstract

**Objective:**

The development of skull base surgery in the past decade has been influenced by advances in visualization techniques; recently, due to such improvements, 3D endoscopes have been widely used. Herein, we address its effect for transnasal endoscopic skull base surgery.

**Methods:**

A total of 63 patients who under endoscopic endonasal surgery (EES) with 3-D endoscope were retrospectively reviewed, including pituitary adenomas, craniopharyngiomas, meningiomas, Rathke’s cleft cysts, and chordomas. According to different lesions, transsellar approach (24 cases), transsphenoidal–transtuberculum approach (14 cases), transclival approach (6 cases), and transpterygoid approach (19 cases) were selected.

**Results:**

Total removal of tumors was achieved in 56 patients (88.9%) and subtotal removal in 7 cases (11.1%). Complications included diabetes insipidus in seven patients (11.1%), cerebrospinal fluid (CSF) leakage in two patients (3.2%), major vascular injury occurred in one patient (1.6%), cranial nerve injury in nine patients (14.3%), and meningitis in two patients (3.2%). There was no mortality in the series. All patients recovered and were back to normal daily life, and no tumor recurrence or delayed CSF leakage was detected during the follow-up (2–13 months, mean 7.59 months).

**Conclusions:**

Via 3D EES, it improved depth perception and preserved important neurovascular tissue when tumors were removed, which is important for improving the operative prognosis.

## Introduction

One of the most challenging areas in neurosurgery is the skull base, which is surrounded by a vast number of crucial neurovascular structures. Furthermore, a tumor’s oncological size, aggressiveness, and irregular expansion make surgical resection of this area highly challenging (1–3). Technical advancements in microscopic development such as the transition from microscopic to endoscopic surgery since the late 1990s benefit from increased efficiency and sufficient surgical field of view that allows more effective surgical treatment of tumors (4–7).

For instance, transnasal techniques and technological advances in the last decade allowed treatments of several skull base lesions through the expanded transsphenoidal approach to the skull base, providing a new approach for treating deep skull base lesions (8). Moreover, neuroendoscopic techniques are widely used in endoscopic endonasal surgery (EES) and are crucial in neurosurgery, otorhinolaryngology, and disorders in the skull base area (9).

Although two-dimensional (2D) high-definition (HD) or ultrahigh-definition (UHD) neuroendoscopes are mostly used nowadays, the lack of depth perception remains an issue. On the other hand, only experienced surgeons can get three-dimensional (3D) depth perception with visual and haptic cues, dynamic movements of the scope, light, shadow, and adequate anatomical knowledge (10, 11). In other words, a relatively steep learning curve is required to overcome such limitations. Nevertheless, following the development and application of 3D camera technology, EES procedures have gradually involved 3D neuroendoscopes with 3D perception in presenting neurovascular tissues (12).

While the use of 3D neuroendoscopes has been reported (13–17), such reports are rare in China. This study presents a retrospective analysis of the results of using 3D EES to treat 63 patients with skull base disorders. A gap in the literature with respect to 3D neuroendoscopic techniques in China, including preliminary experience of the application and advantages of 3D neuroendoscopes, is described.

## Patients and methods

### Patient population

Clinical data of 63 patients who underwent 3D EES from February 2021 to January 2022 were retrospectively analyzed at the Department of Neurosurgery, the First Affiliated Hospital of Nanchang University, China. The cases included 24 males and 39 females with ages ranging from 4 to 72 years (median, 44 years). Clinical data were collected from the patients’ medical records. Among them, 15 cases underwent reoperation. The study conformed to the World Medical Association Declaration of Helsinki ethical principles for medical research involving human subjects. At the same time, each patient involved provided informed consent by signing an informed consent for surgery form.

### Clinical presentation

Pre- and postsurgery evaluations were conducted on the serum levels of free triiodothyronine, free thyroxine, thyroid-stimulating hormone, luteinizing hormone, follicle-stimulating hormone, prolactin, growth hormone, cortisol, and adrenocorticotropic hormone. The preoperative endocrinological evaluation identified 11 patients with hypothyroidism, 5 with decreased adrenocorticotropic hormone, 7 with low testosterone, 4 with hyperprolactinemia, 5 with high-growth hormone, and 8 with low cortisol, while 38 patients showed normal preoperative pituitary hormone function. Furthermore, all patients underwent ophthalmologic examinations (visual acuity and computerized visual field examinations) before and after surgery.

### Neuroradiological evaluation

Preoperative imaging examinations included magnetic resonance imaging (MRI) + enhancement in the sellar area and thin section computed tomography (CT) examination of paranasal sinuses. Preoperative MRI was used to evaluate tumor volume, maximum tumor diameter, and tumor type. Tumor volume was approximated by a modified ellipsoid volume, that is, (A × B × C) × *π*/6, where A–C represent the maximum tumor diameters in each of the three dimensions. Tumor consistency was obtained from preoperative MRI and intraoperative video (Table 1).

**Table 1:** Clinical manifestation and characteristics of patients before surgery.

Variable	No. of cases	%
Total	63	100
Age (years old)	42.6 ± 16.8	
Gender		
Male	24	38.1
Female	39	61.9
Operative history		
Primary	48	76.2
Recurrent	15	23.8
Headache	27	42.9
Visual impairment	35	55.5
Diabetes insipidus	4	6.3
Amenorrhea	10	15.9
Cranial nerve injury		
Abducens nerve	1	1.6
Oculomotor nerve	1	1.6
Hypopituitarism		
Partial hypopituitarism	18	28.6
Panhypopituitarism	5	7.9
Consistency		
Cystic	9	14.3
Solid	44	69.8
Mixed	10	15.9
Maximum diameter		
<3 cm	46	73.0
≥3 cm	17	27.0
Tumor type		
Noninvasive pituitary adenomas	20	31.7
Invasive pituitary adenomas	21	33.3
Craniopharyngiomas	11	17.5
Meningiomas	4	6.3
Rathke’s cleft cyst	4	6.3
Clival chordoma	3	4.8

### Endoscopic equipment

**Figure 1:**
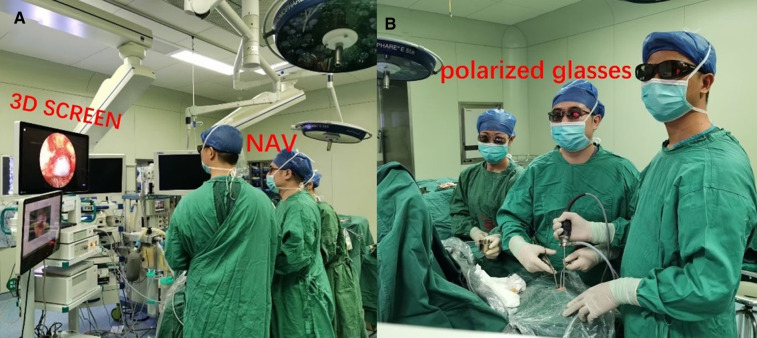
Operating room ergonomics. Position of the device in a comfortable position for surgeon, assistant, and surgical nurses (wearing polarized 3D glasses) to see the 3D video screen directly. (**A**) Placement of the 3D screen (3D SCREEN), neuronavigation (NAV). Note the distance between the surgeon and the 3D screen (at least 2 m), which is necessary for the perception of the 3D image. (**B**) Positioning of surgeon and assistant to have a direct view of the display screen. Operate using the two-person/three-hand technique.

The equipment included a 3D rigid endoscope with 4.0 mm, 0° and 30° (XION, Germany), which is equipped with real 3D and a full-HD monitor. The 3D stereoscopic endoscope with the standard resolution was used on 63 patients. Images were displayed on a 32-inch stereo (dual flat-screen) mirror, 3D, and a full HD monitor (MATRIX *P* Spectar) system that uses a double-coated polarized mirror to overlay right and left images. Polarizing glasses were worn for 3D visualization. All the procedures were performed under the guidance of the Ultrasound Navigation System (BRAINLAB) using MRI or CT data and, in selected cases, utilized the intraoperative portable CT scanner that allowed noncontrast angiography and contrast perfusion scans (Figure 1).

### Surgical procedure

After the application of general anesthesia, all patients were placed in the supine position. The head was slightly extended and rotated 10°–15° to the right and fixed on a rigid, 3-pin Mayfield–Kees skull brace by using image guidance. A rigid 0° endoscope, 18 cm in length and 4 mm in diameter (XION, Germany), was used during the procedure.

All cases were managed using the two-person/three-hand or two-person/four-hand technique. According to different lesions, transsellar approach (24 cases), transsphenoidal–transtuberculum approach (14 cases), transclival approach (6 cases), and transpterygoid approach (19 cases) were selected. During surgery, electrophysiological monitoring, neuronavigation, ultrasound Doppler, and other techniques were applied to the tumor, which enclosed the internal carotid artery or severely damaged the surrounding anatomical structures.

Finally, multilayer skull base reconstruction methods without postoperative lumbar drainage were performed according to intraoperative flow of CSF leakage. Throughout the reconstruction, an iodoform gauze containing aureomycin is used for 7–14 days as a support to prevent graft migration.

### Postoperative management and follow-up

All patients were provided routine prophylactic antibiotics with 100 ml of 0.9% normal saline containing 1.5 g cefuroxime sodium 3 days before surgery within 30–45 min and levofloxacin eye drops (5 mg/ml) in the nose for anti-inflammatory treatment. Patients were asked to undergo a head CT scan within 6 h and MRI enhancement of the sellar area or head within 3 days after surgery to determine the degree of tumor resection and skull base reconstruction. The removal rate for skull base tumors was determined according to intraoperative findings and confirmed by the 3-month follow-up imaging. The gross total resection (GTR) was defined as 100% for tumor resection, 80%–99% subtotal resection (STR), and <80% for partial resection. Postsurgery patients were closely monitored for vital signs, water electrolytes, and hypothalamic-pituitary functions.

Moreover, transnasal neuroendoscopic exploration was performed at 2, 4, and 8 weeks postoperatively to clean the nasal cavity and monitor healing in the nasal mucosa. Finally, after patients are discharged, a regular MRI review method was used to determine the recurrence of primary pathology. Then, postsurgery patients undergo 3-month, 6-month, and annual follow-up imaging and visual assessments; Examinations were further repeated when clinically appropriate.

## Result

GTR was achieved in 56 patients (88.9%), while STR was achieved in 7 (11.1%) patients. Mean tumor volume was 8.18 cm^3^ (range: 0.09–36.65 cm^3^).

The STR patients included one case with pituitary macroadenoma with suprasellar lateral fissure invasion, resulting in a little residue; one case with craniopharyngioma (CP) on the saddle pituitary stalk, resulting in a small residual cyst wall due to thin cyst wall and adhesion of the hypothalamus that could not be separated; one case with cavernous sinus (CS) meningioma where the proximal end of the internal carotid artery at the CS could not be fully exposed; and one case where a hypothalamic-pituitary CP stalk showed residual parts due to the difficult separation of giant calcified plaques from close adhesions on the optic nerve and Circle of Willis. Two cases of recurrent pituitary adenomas (RPA) invading the CS showed a little residual due to significant scar adhesions. One case of a pituitary tumor with invasion of the CS showed a little residue and a small daughter tumor in the direction of the optic nerve.

Preoperative visual impairment was present in 35 cases, improved in 33 cases, and worsened in 2 cases after surgery, including one case of tuberculum sellae meningioma and one case of giant pituitary adenoma. At least 27 cases of preoperative headaches were relieved after an operation, of which 10 cases were from amenorrhea and recovered menstruation. At least five cases of diabetes insipidus (DI) were not significantly relieved through operative therapy. Preoperative oculomotor nerve palsy occurred in one case, relieved considerably after the surgery. One patient showed preoperative symptoms of abducens nerve injury, which was not alleviated after surgery.

The seven cases of postoperative DI include one case of tuberculum sellar meningioma and six cases of CP, all of which were transient DI and improved after drug treatment. Two patients with CS pituitary tumors showed postsurgery epistaxis and recovered by treatment. Two cases of chordoma and two cases of CS RPA developed abducens nerve palsy. Three recurrent cases and one initial case of CS pituitary tumor developed oculomotor nerve palsy. One cases of CS RPA developed trigeminal palsy after surgery. One case of CS RPA developed internal carotid artery injury after surgery, which was treated by electrocoagulation. Two cases of postoperative CP developed CSF leakage and intracranial infection, which were cured by lumbar drainage, CSF leakage repair surgery, and antibiotic treatment. There were no procedure-related deaths.

**Figure 2:**
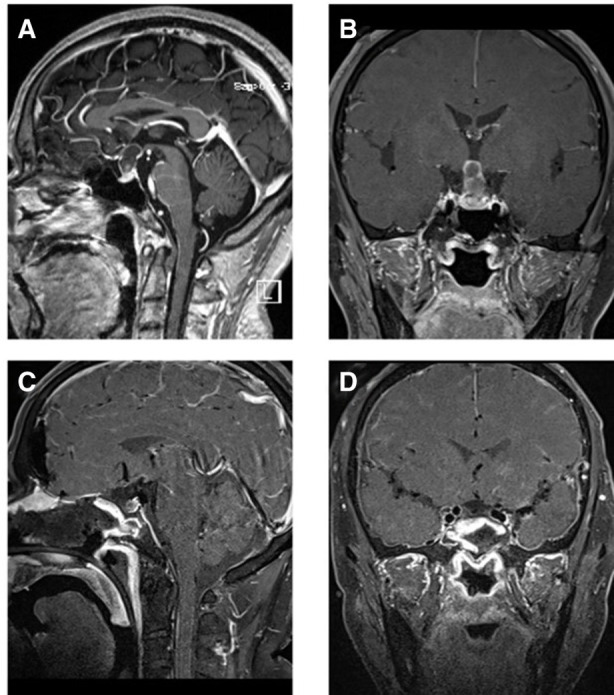
T1-weighted MRI image with contrast enhancement of a suprasellar craniopharyngioma. (**A**) Preoperative sagittal view. (**B**) Preoperative coronal view. (**C**) Postoperative sagittal view. (**D**) Postoperative coronal view. Note in (**C**) and (**D**) the autologous fat employed intradurally to fill the empty space within the suprasellar after tumor removal.

**Figure 3:**
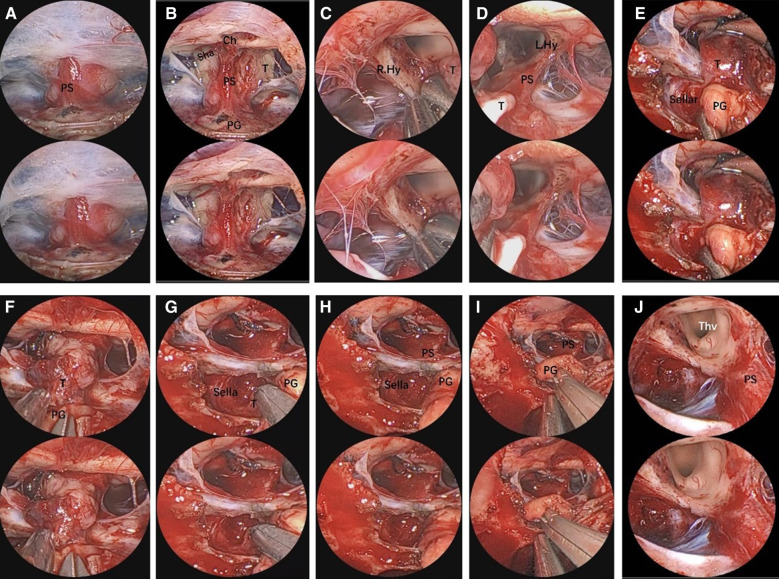
Endoscopic visualization of a suprasellar craniopharyngioma. (**A**) View of the pituitary stalk. (**B**) View of optic nerves, the chiasm, and the anterior complex during extracapsular dissection of tumor. (**C,D**) Close-up view of the pituitary stalk and optic nerves, crucial for tumor dissection. (**E,F**) Anatomical view of the main body of the suprasellar craniopharyngioma. (**G**) A view showing a small part of tumor in the sellar. (**H,I**) View of the PG was pulled and not in placed after surgery. (**J**) Final view of the third ventricle and pituitary stalk after craniopharyngioma removal. The 3D image can be obtained with cross-viewing method. PS, pituitary stalk; PG, pituitary gland; OC, optic chiasm; ON, optic nerve; sha, superior hypophyseal artery; T, tumor; ThV, third ventricle.

**Figure 4:**
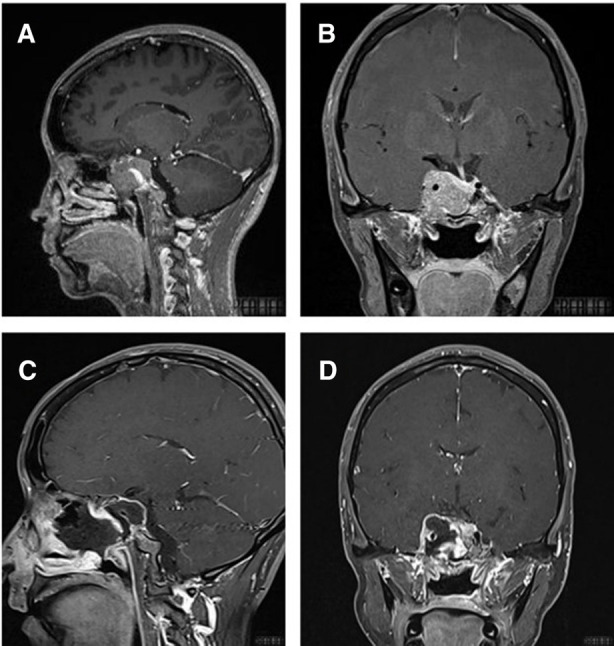
T1-weighted MRI image with contrast enhancement of a cavernous sinus pituitary tumor. (**A**) Preoperative sagittal view. (**B**) Preoperative coronal view. (**C**) Postoperative sagittal view. (**D**) Postoperative coronal view. Note in (**C**) and (**D**) the autologous fat employed intradurally to fill empty space within the sellar after tumor removal.

**Figure 5:**
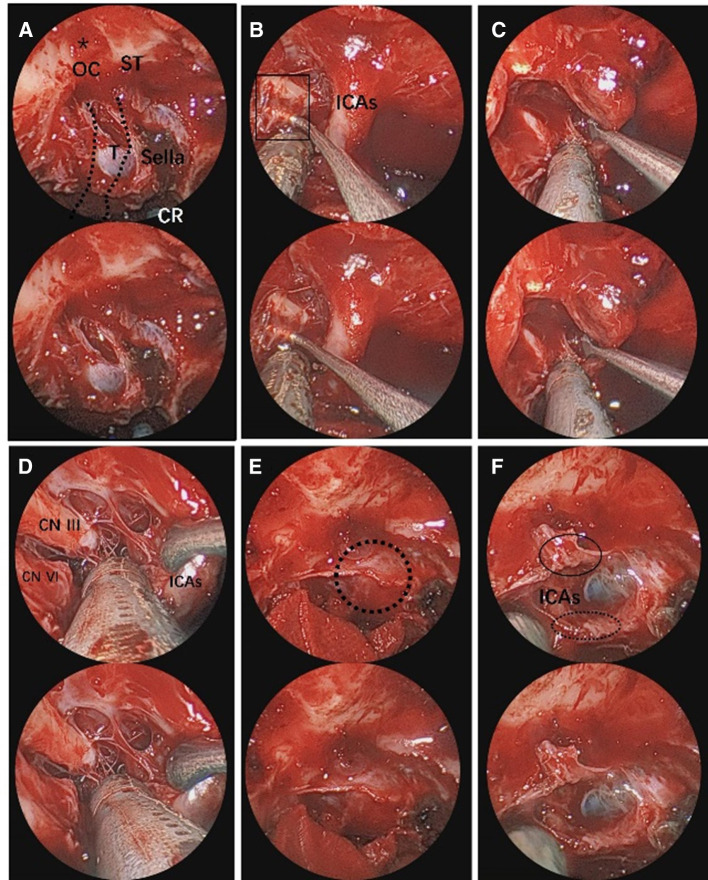
Endoscopic visualization of CS pituitary tumor. (**A**) Exposure of the planum sphenoidale and supraoptic recess (black asterisk). The route of the internal carotid artery. The black asterisk represents the supraoptic recess. (**B**) View of the fiber texture in CS. (**C,D**) Close-up view of the ICAs and nerves, crucial for tumor dissection. (**E**) View of the proximal and distal dural rings of the internal carotid artery (black dotted circle). (**F**) This is the proximal and distal dural ring of the internal carotid artery after resection of the tumor. The black ellipse represents the distal dural ring. The black dotted line ellipse represents the proximal dural ring. The 3D image can be obtained with cross-viewing method. ICAs, intracavernous segment of internal carotid artery; CR, clival recess; CN III, oculomotor nerve; CN VI, abducens nerve; T, tumor; CS, cavernous sinus.

**Figure 6:**
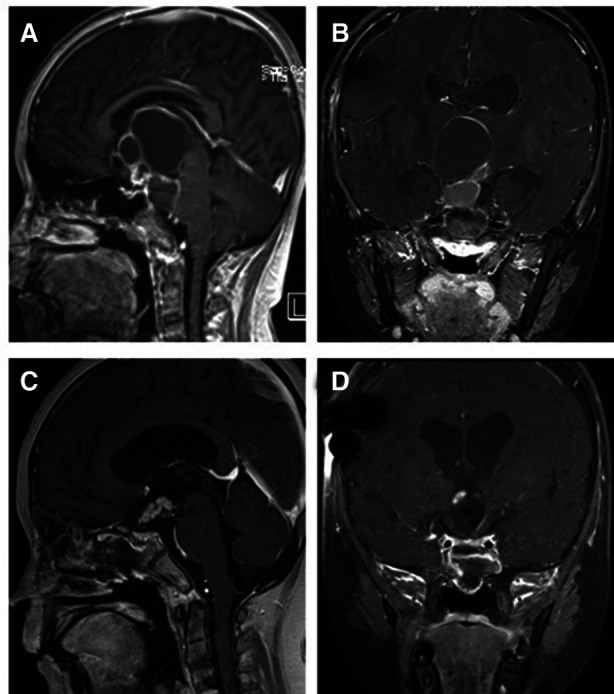
T1-weighted MRI image with contrast enhancement of suprasellar craniopharyngioma. (**A**) Preoperative sagittal view. (**B**) Preoperative coronal view. (**C**) Postoperative sagittal view. (**D**) Postoperative coronal view. Note in (**C**) and (**D**) the autologous fat employed intradurally to fill empty space within the sellar after tumor removal.

**Figure 7:**
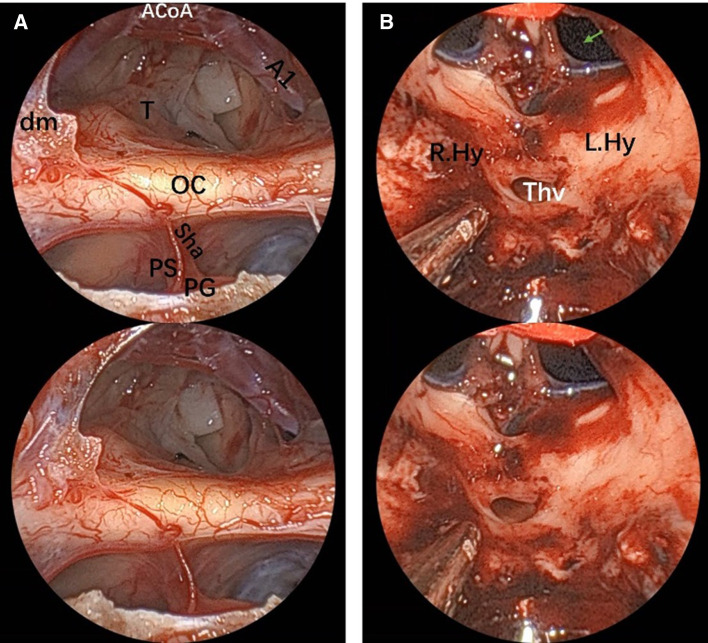
Endoscopic visualization of a suprasellar craniopharyngioma. (**A**) View of optic nerves, the chiasm and the anterior complex during extracapsular dissection of a large suprasellar craniopharyngioma. The tiny vessels adherent to the tumor are clearly demonstrated, which helps perform meticulous dissection maneuver. (**B**) Final view of the third ventricle after craniopharyngioma removal. The 3D image can be obtained with cross-viewing method. PS, pituitary stalk; PG, pituitary gland; OC, optic chiasm; sha, superior hypophyseal artery; T, tumor; dm, dura mater; ThV, third ventricle; green arrow, foramen of Monro; A1, segment of anterior cerebral artery; ACoA, anterior communicating artery, Hy, hypothalamus.

We collected data on tumor types and postoperative complications (shown in Table 2). All cases showed complete healing of nasal mucosa, with rosy color observed by transnasal neuroendoscopic exploration (the important and typical cases are shown in Figures 2–7).

**Table 2:** Major postoperative complications and tumor categories.

Complication	Number and type of tumors
CSF leakage	2 (2 CP)
Transient diabetes insipidus	7 (1 TB sellar meningiomas, 6 CP)
Meningitis	2 (2 CP)
Cranial nerve injury	9 (2 chordoma, 7 cavernous sinus pituitary adenoma)
Epistaxis	2 ( 2 cavernous sinus pituitary adenoma)
Vascular injury	1 (1 cavernous sinus pituitary adenoma)

CSF, cerebrospinal fluid; TB, tuberculum; CP, craniopharyngioma.

All patients received 2–13 months of follow-up examinations (mean: 7.59 months) and MRI reviews that showed no tumor recurrence in all patients. Patients with operative complications during the follow-up period include four cases with abducens nerve palsy, four cases with oculomotor nerve palsy, and one case with trigeminal nerve palsy complications, which completely recovered after rehabilitation treatment and left no relevant sequelae. One case with internal carotid artery injury recovered without developing pseudoaneurysm. One case with vision loss showed no continuing aggravation. Seven DI cases recovered to normal status after medication. One case with preoperative symptoms of oculomotor nerve palsy recovered normal function.

Finally, all patients maintained normal endocrine levels and returned to normal life. Moreover, there were no cases of deaths or delayed CSF leakage during the follow-up period (Table 3).

**Table 3:** Preoperative, postoperative, and follow-up statuses in all patients.

Statuses	Total	Preoperative	Postoperative			Follow-up Normalized
			Improved	Unchanged	Worsened	
Endocrinological						
Normal pituitary function	38	38	—	35 (92.1%)	3 (7.9%)	38 (100%)
Partial hypopituitarism	18	18	13 (72.2%)	4 (22.2%)	1 (5.5%)	18 (100%)
Panhypopituitarism	5	5	5 (100%)	0 (0%)	0 (0%)	5 (100%)
Preop DI	5	5	0 (0%)	5 (100%)	0 (0%)	0 (0%)
New cases of DI	7	—	—	—	—	7 (100%)
Hyperprolactinemia	4	4	4 (100%)	0 (0%)	0 (0%)	4 (100%)
High GH	5	5	5 (100%)	0 (0%)	0 (0%)	5 (100%)
Clinical symptoms						
Headache	27	27	27 (100%)	0 (0%)	0 (0%)	27 (100%)
Visual impairment	35	35	33 (94.3%)	0 (0%)	2 (5.7%)	33 (94.3%)
Visual normal	28	28	0 (0%)	28 (100%)	0 (0%)	28 (100%)
Amenorrhea	4	4	4 (100%)	0 (0%)	0 (0%)	4 (100%)
Preop CN palsy	2	2	1 (50%)	1 (50%)	0 (0%)	2 (50%)
New cases of CN palsy	9	—	—	—	—	9 (100%)

DI, diabetes insipidus; GH, growth hormone; CN, cranial nerve.

## Discussion

The most challenging types of skull base surgery involve pituitary tumors, one of the most common tumors in the sellar region and include invasive and noninvasive pituitary tumors. Moreover, CS invasion is associated with high surgical risks and recurrence rates, STR and endocrine remission, and the need for adjuvant therapy (18, 19). To address these issues, Cushing suggested a transsphenoidal surgical approach in 1907 (20). Through the exploration of surgeons and the development of endoscopic techniques, EES has become one of the primary approaches for treating skull base tumors (21–23). As surgeons gained EES experience and development, they advanced into novel territories such as complex, skull base tumors (e.g., CS invasion tumor), which were once considered inoperable.

Due to the increasing use and difficulty of EES cases, the 2D neuroendoscopes became insufficient to meet surgical demands. Further development of new devices and instruments for EES, from the initial single-chip endoscopic camera with bright and dark, single-color displays to the three-chip, HD, and UHD 2D neuroendoscopes, can help in enhancing neurosurgery technology.

Because of the 3D nature of human anatomy, depth perception is essential to all surgical procedures. Since the widely used 2D neuroendoscopes cannot meet surgical requirements, developing 3D neuroendoscopes with 3D visibility is significant (24).

With its 3D visualization advantages, the 3D endoscope was first applied in laparoscopic surgery, then developed (14–16, 25) and gradually applied to other surgical fields. However, its development in neurosurgery was not ideal due to the large size of the early 3D endoscope, which had a 4.9 mm tip diameter.

With developments in 3D and small-aperture camera technology, the new generation of 4.0 mm 3D HD neuroendoscope provides advantages such as better depth perception and improved hand–eye coordination for surgeons, and is widely used in skull base surgery, particularly in the treatment of deep brain lesions. In addition, this new neuroendoscope provides better resolution of tiny neurovascular structures so that surgeons can assess more accurately the tissue structure and the distances between lesions and critical neurovascular structures (13, 26–28). Due to these advantages, the 3D neuroendoscope has been increasingly used in EES. Moreover, research studies confirm its practical clinical value in different treating skull base lesions.

Although studies have shown that 3D endoscopic techniques have been developed earlier in other countries, it has not been used as widely as 2D endoscopic devices due to the high cost of 3D endoscopic devices when they were initially developed, the high cost of the procedure, the lack of user experience, and adverse visual effects such as visual fatigue and lack of 3D depth perception (6, 29, 30).

However, our 3D technology exploration is relatively late. Now, fairly well-developed 3D equipment is available, along with surgeons experienced in endoscopic techniques. Moreover, the lower procedural cost and surgeons with more experience in endoscopic techniques can help reduce or avoid the adverse effects associated with 3D endoscopes, such as visual fatigue and imperfect depth perception. In other words, the use of 3D EES can be promoted. Although this study found complications such as cranial nerve injury and DI, cranial nerve palsy and endocrine levels recovered to normal due to rehabilitation. In particular, the depth perception obtained by 3D endoscopes allowed precise dissection, thus protecting the neurovascular tissue during CS pituitary tumors and CP surgery.

This report describes the use of 3D neuroendoscopes (XION, Germany) in EES as follows: (1) The layout of the endoscopic surgery room is consistent with previous EES use of a 2D neuroendoscope. However, the correct distance of the surgeons should be at least 2 m from the 3D screen or at least 1 m farther than when using a 2D neuroendoscope. This distance is crucial for viewing the 3D image and reducing visual fatigue to avoid the effects of long-term 3D use on the surgeon’s vision (31). (2) The 3D system can overcome the major drawbacks of lens contamination and visual degradation when operating in a narrow nasal cavity (32). During the tumor dissection, 3D endoscopy is most helpful in understanding the surgical anatomy (33). Therefore, better knowledge of the 3D transnasal skull base anatomy allows a more selective dissection of the structure of the skull base, even in the CS, during EES. (3) After completing the surgical approach, removing lesion in the open field of vision can best display the 3D visual effect and the lens is seldom polluted. In other words, the 3D depth perception is effective in reducing the incidence of complications. The HD resolution clearly differentiates a neural subdural lesion from adjoining blood vessels and diseased vs. healthy peripheral nerve blood vessels (17, 31, 34). (4) Although the 3D neuroendoscope has only 0° and 30° endoscopes and no 45° and 70° endoscopes, in most cases, a 30° 3D endoscope allows surgeons to see skull base corners and perform surgical procedures under direct vision. (5) Surgeons with rich EES experience and long-term use of the 2D neuroendoscope will only need a very short time to fully adapt to the stereoscopic effect of the 3D neuroendoscope. Moreover, junior doctors who have just started with EES do not need a long learning curve (35, 36).

The total resection rate was 88.9% (56/63) in this study, similar to the data reported in the literature. Moreover, the surgical results are reasonable when compared with data retrieved from recent literature reports (Table 4) (27, 29, 30, 37–40).

**Table 4:** Literature review of complications of 3D EES.

Authors (year)	No. of patient	GTR (N/%)	Cerebrospinal fluid leakage (N/%)	Meningitis (N/%)	Vascular injury (N/%)	Epistaxis (N/%)	Diabetes insipidus (N/%)	Hypopituitarism (N/%)
Pennacchietti et al. (2016) (37)	104	73 (70.1)	5 (4.8)	N P	2 (1.9)	1 (1.9)	6 (5.7)	17 (16.3)
Tabaee et al. (2009) (38)	13	10 (76.9)	0	0	0	0	N P	N P
Kari et al. (2010) (27)	26	N P	1 (3.8)	N P	N P	N P	5 (19.2)	1 (3.8)
Felisati et al. (2013) (29)	10	6 (60)	2 (20)	2 (20)	N P	N P	2 (20)	5 (50)
Haidari et al. (2018) (39)	116	87 (75)	11 (9.5)	4 (3.4)	N P	7 (6.0)	N P	11 (9.5)
Barkhoudarian et al. (2013) (40)	65	N P	1 (1.6)	N P	N P	2 (3.1)	1 (1.6)	N P
Catapano et al. (2016) (30)	70	50 (71.4)	5 (7.1)	1 (1.4)	N P	N P	9 (12.9)	9 (12.9)
Present study	63	56 (88.9)	2 (3.2)	2 (3.2)	1 (1.6)	2 (3.2)	7 (11.1)	4 (6.3)

EES, endoscopic endonasal surgery; GTR, gross total resection; N P, Not Reported.

The preceding literature review and this study conclude that the advantages of 3D neuroendoscope in EES include improved depth perception, identification of deep anatomical structures, and enhanced surgery safety. Patient complications, such as DI, nerve injury, and CSF leakage, were related to lesions but not to the 3D neuroendoscopic technique or the surgical instruments. While the significant effects of 3D vision are evident, further controlled studies with more patients are necessary to assess the objective significance of 3D visualization in EES.

## Limitations of this study

One limitation of this study is the lack of a controlled study on 2D and 3D endoscopic surgical outcomes. The potential value of 3D stereoscopic visualization is evident, explaining why the next research focus should be on assessing the objective significance of 3D visualization techniques in transnasal skull base surgery through a controlled study with an appropriate sample.

## Conclusions

The 3D endoscope can overcome the principal limit of the 2D endoscope: the lack of depth perception. Using the advantages of 3D neuroendoscope, it is indeed conducive to more stereoscopic and subtle perception of deep anatomical structures by the physician during EES, overcoming the major drawbacks of traditional 2D neuroendoscope, facilitating hand–eye coordination, discerning the important peripheral neurovascular structures during tumor resection, and improving the safety and efficacy of surgery. We point out that the 3D endoscope is a concrete and promising development tool for EES.

## Data Availability

The original contributions presented in the study are included in the article/Supplementary Material, further inquiries can be directed to the corresponding author.

## References

[1] Abu-Ghanem S Shilo S Yehuda M Abergel A Safadi A Fliss DM. Anterior skull base surgery in the 21st century: the role of open approaches. Adv Otorhinolaryngol. (2020) 84:56–67. 10.1159/000457925 32731242

[2] Castelnuovo P Battaglia P Bignami M Ferreli F Turri-Zanoni M Bernardini E Endoscopic transnasal resection of anterior skull base malignancy with a novel 3D endoscope and neuronavigation. Acta Otorhinolaryngol Ital. (2012) 32(3):189–91. 22767985 PMC3385062

[3] Cavallo LM Somma T Solari D Iannuzzo G Frio F Baiano C Endoscopic endonasal transsphenoidal surgery: history and evolution. World Neurosurg. (2019) 127:686–94. 10.1016/j.wneu.2019.03.048 31266131

[4] Emanuelli E Zanotti C Munari S Baldovin M Schiavo G Denaro L. Sellar and parasellar lesions: multidisciplinary management. Acta Otorhinolaryngol Ital. (2021) 41(Suppl. 1):S30–41. 10.14639/0392-100X-suppl.1-41-2021-03 34060518 PMC8172107

[5] Fletcher AM Marentette L. Anterior skull-base surgery: current opinion. Curr Opin Otolaryngol Head Neck Surg. (2014) 22(4):322–5. 10.1097/MOO.0000000000000073 24991746

[6] Vasudevan K Saad H Oyesiku NM. The role of three-dimensional endoscopy in pituitary adenoma surgery. Neurosurg Clin N Am. (2019) 30(4):421–32. 10.1016/j.nec.2019.05.012 31471049

[7] Wagenmann M Scheckenbach K Kraus B Stenin I. [Complications of anterior skull base surgery]. HNO. (2018) 66(6):438–46. 10.1007/s00106-018-0508-3 29740678

[8] Roth JA Seljeskog EL Duvall AJ 3rd Long DM. Transnasal transsphenoidal approach to the sella. Laryngoscope. (1977) 87(1):47–57. 10.1288/00005537-197701000-00006 63889

[9] Maroon JC. Skull base surgery: past, present, and future trends. Neurosurg Focus. (2005) 19(1):E1. 10.3171/foc.2005.19.1.2 16078812

[10] Baussart B Declerck A Gaillard S. Mononostril endoscopic endonasal approach for pituitary surgery. Acta Neurochir (Wien). (2021) 163(3):655–9. 10.1007/s00701-020-04542-z 32862300

[11] Al Kadah B Bumm K Charalampaki P Schick B. [First experience in endonasal surgery using a new 3D-chipendoscope]. Laryngorhinootologie. (2012) 91(7):428–33. 10.1055/s-0032-1309051 22581663

[12] Wang AJ Zaidi HA Laws ED Jr. History of endonasal skull base surgery. J Neurosurg Sci. (2016) 60(4):441–53. 27273318

[13] Abarca-Olivas J Monjas-Canovas I Lopez-Alvarez B Lloret-Garcia J Sanchez-del Campo J Gras-Albert JR [Three-dimensional endoscopic endonasal study of skull base anatomy]. Neurocirugia (Astur). (2014) 25(1):1–7. 10.1016/j.neucir.2013.02.009 24447642

[14] Inoue D Yoshimoto K Uemura M Yoshida M Ohuchida K Kenmotsu H Three-dimensional high-definition neuroendoscopic surgery: a controlled comparative laboratory study with two-dimensional endoscopy and clinical application. J Neurol Surg A Cent Eur Neurosurg. (2013) 74(6):357–65. 10.1055/s-0033-1345100 23888482

[15] Kawanishi Y Fujimoto Y Kumagai N Takemura M Nonaka M Nakai E Evaluation of two- and three-dimensional visualization for endoscopic endonasal surgery using a novel stereoendoscopic system in a novice: a comparison on a dry laboratory model. Acta Neurochir (Wien). (2013) 155(9):1621–7. 10.1007/s00701-013-1757-2 23686635

[16] Liang H Liang W Lei Z Liu Z Wang W He J Three-dimensional versus two-dimensional video-assisted endoscopic surgery: a meta-analysis of clinical data. World J Surg. (2018) 42(11):3658–68. 10.1007/s00268-018-4681-z 29946785

[17] Marcus HJ Hughes-Hallett A Cundy TP Di Marco A Pratt P Nandi D Comparative effectiveness of 3-dimensional vs 2-dimensional and high-definition vs standard-definition neuroendoscopy: a preclinical randomized crossover study. Neurosurgery. (2014) 74(4):375–80, discussion 380–1. 10.1227/NEU.0000000000000249 24220007 PMC4053590

[18] Wu X Xie SH Tang B Yang YQ Yang L Ding H Pituitary adenoma with posterior area invasion of cavernous sinus: surgical anatomy, approach, and outcomes. Neurosurg Rev. (2021) 44(4):2229–37. 10.1007/s10143-020-01404-1 33006012

[19] Doglietto F Lauretti L Frank G Pasquini E Fernandez E Tschabitscher M Microscopic and endoscopic extracranial approaches to the cavernous sinus: anatomic study. Neurosurgery. (2009) 64(5 Suppl 2):413–21, discussion 421–2. 10.1227/01.NEU.0000338943.08985.73 19404119

[20] Cushing H III. Partial hypophysectomy for acromegaly: with remarks on the function of the hypophysis. Ann Surg. (1909) 50(6):1002–17. 10.1097/00000658-190912000-00003 17862444 PMC1407483

[21] Verillaud B Bresson D Sauvaget E Mandonnet E Georges B Kania R Endoscopic endonasal skull base surgery. Eur Ann Otorhinolaryngol Head Neck Dis. (2012) 129(4):190–6. 10.1016/j.anorl.2011.09.004 22321910

[22] Schwartz TH Morgenstern PF Anand VK. Lessons learned in the evolution of endoscopic skull base surgery. J Neurosurg. (2019) 130(2):337–46. 10.3171/2018.10.JNS182154 30717035

[23] Munson PD Moore EJ. Pediatric endoscopic skull base surgery. Curr Opin Otolaryngol Head Neck Surg. (2010) 18(6):571–6. 10.1097/MOO.0b013e3283401fdc 20962646

[24] Fraser JF Allen B Anand VK Schwartz TH. Three-dimensional neurostereoendoscopy: subjective and objective comparison to 2d. Minim Invasive Neurosurg. (2009) 52(1):25–31. 10.1055/s-0028-1104567 19247901

[25] Sinha RY Raje SR Rao GA. Three-dimensional laparoscopy: principles and practice. J Minim Access Surg. (2017) 13(3):165–9. 10.4103/0972-9941.181761 27143695 PMC5485803

[26] Stokken JK Halderman A Recinos PF Woodard TD Sindwani R. Strategies for improving visualization during endoscopic skull base surgery. Otolaryngol Clin North Am. (2016) 49(1):131–40. 10.1016/j.otc.2015.09.008 26614833

[27] Kari E Oyesiku NM Dadashev V Wise SK. Comparison of traditional 2-dimensional endoscopic pituitary surgery with new 3-dimensional endoscopic technology: intraoperative and early postoperative factors. Int Forum Allergy Rhinol. (2012) 2(1):2–8. 10.1002/alr.20036 22311834

[28] Patel SK Kashyrina O Duru S Miyabe M Lim FY Peiro JL Comparison of two- and three-dimensional endoscopic visualization for fetal myelomeningocele repair: a pilot study using a fetoscopic surgical simulator. Childs Nerv Syst. (2021) 37(5):1613–21. 10.1007/s00381-020-04999-4 33392653

[29] Felisati G Lenzi R Pipolo C Maccari A Messina F Revay M Endoscopic expanded endonasal approach: preliminary experience with the new 3d endoscope. Acta Otorhinolaryngol Ital. (2013) 33(2):102–6. 23853400 PMC3665380

[30] Catapano G de Notaris M Di Maria D Fernandez LA Di Nuzzo G Seneca V The use of a three-dimensional endoscope for different skull base tumors: results of a preliminary extended endonasal surgical series. Acta Neurochir (Wien). (2016) 158(8):1605–16. 10.1007/s00701-016-2847-8 27278644

[31] Kikuchi D Kaise M Nomura K Toba T Kuribayashi Y Tanaka M Feasibility study of the three-dimensional flexible endoscope in endoscopic submucosal dissection: an ex vivo animal study. Digestion. (2017) 95(3):237–41. 10.1159/000468924 28365684

[32] Bickerton R Ahmed S Kholief A Nassimizadeh AK. Breadth and depth: three-dimensional endoscopic field of view: two-dimensional versus three-dimensional endoscopic field of view. World Neurosurg. (2019) 127:e717–21. 10.1016/j.wneu.2019.03.247 30947003

[33] Ogino-Nishimura E Nakagawa T Sakamoto T Ito J. Efficacy of three-dimensional endoscopy in endonasal surgery. Auris Nasus Larynx. (2015) 42(3):203–7. 10.1016/j.anl.2014.10.004 25459496

[34] Wasserzug O Margalit N Weizman N Fliss DM Gil Z. Utility of a three-dimensional endoscopic system in skull base surgery. Skull Base. (2010) 20(4):223–8. 10.1055/s-0030-1247630 21311614 PMC3023316

[35] Egi H Hattori M Suzuki T Sawada H Kurita Y Ohdan H. The usefulness of 3-dimensional endoscope systems in endoscopic surgery. Surg Endosc. (2016) 30(10):4562–8. 10.1007/s00464-016-4793-1 26895893

[36] Roth J Singh A Nyquist G Fraser JF Bernardo A Anand VK Three-dimensional and 2-dimensional endoscopic exposure of midline cranial base targets using expanded endonasal and transcranial approaches. Neurosurgery. (2009) 65(6):1116–28, discussion 1128–30. 10.1227/01.NEU.0000360340.85186.7A 19934971

[37] Pennacchietti V Garzaro M Grottoli S Pacca P Garbossa D Ducati A Three-dimensional endoscopic endonasal approach and outcomes in sellar lesions: a single-center experience of 104 cases. World Neurosurg. (2016) 89:121–5. 10.1016/j.wneu.2016.01.049 26836697

[38] Tabaee A Anand VK Fraser JF Brown SM Singh A Schwartz TH. Three-dimensional endoscopic pituitary surgery. Neurosurgery. (2009) 64(5 Suppl 2):288–93, discussion 294–5. 10.1227/01.NEU.0000338069.51023.3C 19404107

[39] Hajdari S Kellner G Meyer A Rosahl S Gerlach R. Endoscopic endonasal surgery for removal of pituitary adenomas: a surgical case series of treatment results using different 2- and 3-dimensional visualization systems. World Neurosurg. (2018) 119:e80–6. 10.1016/j.wneu.2018.07.018 30010078

[40] Barkhoudarian G Del Carmen Becerra Romero A Laws ER. Evaluation of the 3-dimensional endoscope in transsphenoidal surgery. Neurosurgery. (2013) 73:ons74–ons79. 10.1227/NEU.0b013e31828ba962 23407288

